# Stem Cells as New Agents for the Treatment of Infertility: Current and Future Perspectives and Challenges

**DOI:** 10.1155/2014/507234

**Published:** 2014-04-14

**Authors:** Vladislav Volarevic, Sanja Bojic, Jasmin Nurkovic, Ana Volarevic, Biljana Ljujic, Nebojsa Arsenijevic, Majlinda Lako, Miodrag Stojkovic

**Affiliations:** ^1^Centre for Molecular Medicine and Stem Cell Research, Faculty of Medical Sciences, University of Kragujevac, 69 Svetozara Markovica Street, 34000 Kragujevac, Serbia; ^2^Institute of Genetic Medicine, International Centre for Life, Newcastle University, Central Parkway, Newcastle upon Tyne NE1 3BZ, UK; ^3^Spebo Medical, 16 Norvezanska Street, 16000 Leskovac, Serbia

## Abstract

Stem cells are undifferentiated cells that are present in the embryonic, fetal, and adult stages of life and give rise to differentiated cells that make up the building blocks of tissue and organs. Due to their unlimited source and high differentiation potential, stem cells are considered as potentially new therapeutic agents for the treatment of infertility. Stem cells could be stimulated *in vitro* to develop various numbers of specialized cells including male and female gametes suggesting their potential use in reproductive medicine. During past few years a considerable progress in the derivation of male germ cells from pluripotent stem cells has been made. In addition, stem cell-based strategies for ovarian regeneration and oocyte production have been proposed as future clinical therapies for treating infertility in women. In this review, we summarized current knowledge and present future perspectives and challenges regarding the use of stem cells in reproductive medicine.

## 1. Introduction

Nearly 72.4 million people or 15% of couples experience fertility problems [1]. For couples and clinicians, a diagnosis of infertility signals the start of investigations and possible treatment. Infertility, defined as failure to conceive a clinically detectable pregnancy after >12 months of unprotected intercourse, is a common condition, reported by 1 in 6 couples [1, 2]. As infertility is a heterogeneous condition, caused by various underlying pathologies, it is possible that some of the mechanisms leading to infertility also play a role in the etiology of this outcome [3–5]. In recent years, several advancements have been made in assisted reproduction treatment and now more than 80% of couples experiencing infertility issues can conceive a child [6].

Due to their unlimited source and high differentiation potential, stem cells are considered as potentially new therapeutic agents for the treatment of infertility. In this review, we will summarize current knowledge regarding the use of stem cells in reproductive medicine.

## 2. Stem Cells: A Novel Hope in Cell-Based Therapy

Stem cells are undifferentiated cells that are present in the embryonic, fetal, and adult stages of life and give rise to differentiated cells that are building blocks of tissue and organs (Table 1). In the postnatal and adult stages of life, tissue-specific stem cells are found in differentiated organs and are instrumental in repair following injury to the organ. The major characteristics of stem cells are (a) self-renewal (the ability to extensively proliferate), (b) clonality (usually arising from a single cell), and (c) potency (the ability to differentiate into different cell types) [7, 8]. Totipotent or omnipotent cells are the most undifferentiated cells and are found in early development. A fertilized oocyte and the cells of the first two divisions are totipotent cells, as they differentiate into both embryonic and extraembryonic tissues, thereby forming the embryo and the placenta [9]. Pluripotent stem cells are able to differentiate into cells that arise from the 3 germ layers—ectoderm, endoderm, and mesoderm—from which all tissues and organs develop [10]. Commonly, stem cells are derived from two main sources: early embryos (embryonic stem cells (ESCs)) and adult tissue (adult stem cells).

ESCs are pluripotent stem cells derived from the inner cell mass of the blastocyst [11]. The essential characteristics of ESCs include derivation from the preimplantation embryo, prolonged proliferation in their pluripotent state, and stable developmental potential to form derivatives of all three embryonic germ layers [11].

Mesenchymal stem cells (MSCs) are one of the most common adult, multipotent stem cells [12]. They can be derived from a variety of tissues including bone marrow, adipose tissue, bone, Wharton's jelly, umbilical cord blood, and peripheral blood [13]. MSCs are adherent to cell culture dishes and are characterized by specific surface cell markers. MSCs show variable levels of expression of several molecules, CD105 (SH2), CD73 (SH3/4), stromal antigen 1, CD44, CD166 (vascular cell adhesion molecule), CD54/CD102 (intracellular adhesion molecule), and CD49 (very late antigen), and lack the expression of surface markers characteristic for HSCs (CD14, CD34, CD45, and CD11a/LFA-1), erythrocytes (glycophorin A), and platelet and endothelial cell (CD31). MSCs are able to differentiate into mesoderm-derived tissue such as adipose tissue, bone, cartilage, and muscle [13–16]. Recently, MSCs were differentiated into neuronal tissue which is derived from the ectoderm. This is an example of transdifferentiation, that is, when a cell from one germ layer (mesoderm) differentiates into neuronal tissue (ectoderm) [17].

Stem cells can also be derived from extraembryonic tissues (amnion, chorion, placenta, and umbilical cord) [18]. Amnion and chorion contain stromal cells that display characteristics and differentiation potential similar to bone marrow-derived MSCs and are able to differentiate into adipocytes, endothelial cells, hepatocytes, osteocytes, myocytes, and neurons [7, 18]. Placental-derived stem cells have the capacity to differentiate into ectodermal, mesodermal, and endodermal cell types, while umbilical cord matrix stem cells, after transplantation, enhanced muscle regeneration in mouse model of severe muscle damage and promoted blood vessel formation and neurological function in animal models of ischaemic brain disease [18]. The main advantage of stem cells derived from extraembryonic tissues is the efficient isolation from tissues normally discarded at birth avoiding ethical concerns that plague the isolation of human embryonic stem cells [7] (Table 2).

Recently, Takahashi and Yamanaka [19] generated pluripotent cells by reprogramming somatic cells. These cells are called induced pluripotent stem cells (iPSCs) and share similar characteristics with ESCs: exhibiting morphology of ESCs, expressing ESCs markers, having normal karyotype, expressing telomerase activity, and maintaining the developmental potential to differentiate into derivatives of all three primary germ layers. Thus, iPSCs are adult cells that have been genetically reprogrammed to an embryonic stem cell-like state by being forced to express genes and factors important for maintaining the defining properties of embryonic stem cells [20]. Transplantation of stem cells or their derivatives into respective tissues or organs is considered as one of the most promising remedies for many incurable diseases. Unfortunately, immune compatible cells are hardly obtainable for any given patient because of the specificity and complexity of human immune system. In this regard, induced pluripotent stem cells (iPSCs) and gene editing technologies are believed to offer an unprecedented solution for obtaining sufficient healthy autologous cells [21]. However, it should be emphasized that, despite numerous technical advances in the reprogramming technology, iPSCs apart from a very small number of ongoing clinical studies are not yet ready for transplanting into patients. Relatively little is known about iPSCs molecular and functional equivalence to hESCs and careful analysis of the genomic and epigenomic integrity of human iPSCs is required before their therapeutic use.

Stem cells could be stimulated* in vitro* to develop various numbers of specialized cells including male and female gametes suggesting their potential use in reproductive medicine.

## 3. Stem Cells Are Novel and Unlimited Source for Male Gametes: True or False?

During past few years a considerable progress in the derivation of male germ cells from pluripotent stem cells has been made [22–24]. These studies provide a desirable experimental model for elucidating underlying molecular mechanism of male germ cell development and potential strategies for producing haploid germ cells for the treatment of male infertility.

Spermatogenesis is a complex process by which spermatogonial stem cells (SSC) self-renew and differentiate into haploid spermatozoa. In mammals, this process takes place in the seminiferous tubules of testis, which provide a functional niche for male germ cells [25] and involve three major stages: mitosis, meiosis, and spermiogenesis [24]. Errors at any stage of spermatogenesis can result in subfertility and infertility [26].

SSC reside in adult testis and maintain spermatogenesis and continual sperm production throughout a male's lifespan [27]. SSC are diploid cells that originate from less differentiated primordial germ cells that migrate to the gonadal ridges during embryogenesis [28]. SSC can be found in the seminiferous tubule, lying near to the basement membrane [29]. Several markers could be used for the identification and isolation of SSC: spermatogonia-specific marker Stra8 for mouse SSC [30, 31], thymocyte antigen 1 (Thy-1), CD9, stage-specific embryonic antigen-4 (SSEA4), *β*1 and *α*6 integrins for rat SSC [32], SSEA4, and G-protein coupled receptor 125 (GPR125) for human SSC [33]. SSC are a potential tool for the treatment of male infertility due to their ability to differentiate into male gametes* in vitro* and capacity to restore male fertility* in vivo* [34, 35]. SSC are adult stem cells, but SSC-derived cells, called multipotent adult germline stem cells (maGSC), have differentiation potential similar to ESCs.* In vitro*, maGSC are able to spontaneously differentiate into derivatives of all embryonic germ layers and are able to generate teratomas after transplantation in immunodeficient mice [31]. Nolte and coworkers showed that maGSC are able to undergo meiosis and form haploid male germ cells* in vitro* [30]. An important breakthrough for SSC-mediated spermatogenesis was made by Hermann and coworkers [35]. They showed that autologous and allogeneic SSC transplantations into the testes of adult and prepubertal recipient macaques, which were rendered infertile with alkylating chemotherapy, regenerate spermatogenesis resulting in production of functional sperm. These results strongly indicate SSC transplantation as a novel and successful therapeutic tool for male infertility caused by chemotherapy before puberty [35]. Although SSC seem to be a good candidate for the stem cell-based therapy of male infertility, a low concentration of SSCs in mammal testis and challenges associated with protocols for their isolation, identification, and culturing have to be addressed before their clinical use [29].

Hübner et al. first reported the successful derivation of gametes from mouse embryonic stem cells (ESCs)* in vitro* [36]. Afterwards, different studies with mouse ESCs have shown the ability to make functional spermatozoa [37, 38] capable of giving rise to live offspring after use of intracytoplasmatic injection [37]. Differentiation of male germ cells from human ESC has also been demonstrated [39–43]. Similarly, studies with human ESCs have revealed the ability to differentiate* in vitro* into advanced spermatogenic stages, including round spermatids which are not capable of fertilizing oocytes in high-order mammals [22, 23].

Besides the fact that ESCs are genetically unrelated to the patient in need of fertilization treatment, the isolation of human ESCs is ethically controversial because it involves the destruction of human embryos. The significant breakthrough in stem cell biology, a discovery of patient-specific induced pluripotent stem cells (iPSCs), may overcome these issues. Recently, several studies have reported that both mouse iPSCs [24, 44, 45] and human iPSCs can differentiate into male germ cells [20, 21].

It has been verified that mouse iPSCs can form functional spermatozoa [46, 47]. Functional assays have shown that spermatozoa generated from iPSCs were capable of fertilizing the oocytes after intracytoplasmatic injection and giving rise to fertile offspring following embryo transfer [46]. So far, functional male gametes from human iPSCs have not been obtained.

There are two possible approaches in generating of male germ cells from pluripotent stem cells:* in vitro* differentiation into advanced, haploid cell products [20, 21] or combined* in vitro* differentiation and* in vivo* transplantation [24, 48]. Generally, there are two methods to produce male gametes from the pluripotent stem cells* in vitro* [20]: the monolayer differentiation and the embryoid body (EB) formation [24, 44]. Direct differentiation on monolayers of human fibroblast ensures more consistent differentiation results compared with EB formation [20].

**Figure 1:**
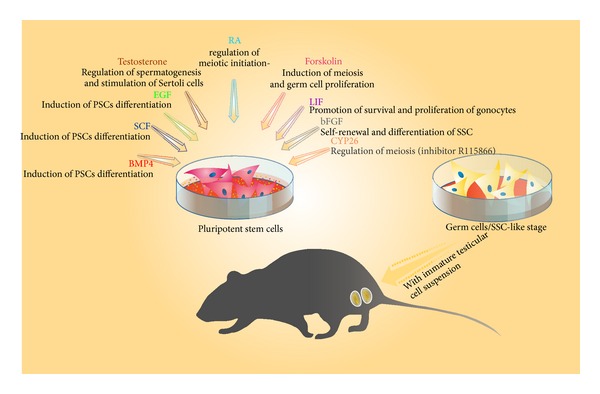
Stem cell-derived male gametes. Several growth factors and cytokines are used for* in vitro* differentiation of pluripotent cells into male gametes/SSC-like cells. The transplantation of stem cell-derived SSC-like cells in sterile mice results in proper spermatogenesis.

Different growth factors or cytokines could induce pluripotent stem cells into germ cells* in vitro* (Figure 1), such as bone morphogenetic protein 4, stem cell factor, epidermal growth factor, and forskolin, but most of* in vitro* differentiation protocols include retinoic acid (RA) induction [46, 49, 50]. It has been shown that RA, an active derivate of vitamin A, regulates the timing of meiotic initiation in mice [50, 51]. Some protocols include combination of RA and testosterone [45] or subsequent exposure to differentiation cocktail containing forskolin, human leukemia inhibiting factor (LIF), bFGF, and CYP26 inhibitor R115866 [22]. Testosterone is required for spermatogenesis* in vivo* and stimulates Sertoli cells to produce different growth factors, including stem cell factor that promotes germ cell differentiation [52]. Forskolin is involved in meiosis induction [53] and induces germ cell proliferation by activation of cyclic adenosine monophosphate [54]. LIF promotes survival and proliferation of gonocytes [55], while bFGF helps balancing self-renewal and differentiation of SSC [56]. R115866 acts by suppressing the inhibitory effects of CYP26 on STRA8, the meiosis regulator gene [51].

In some studies combination of* in vitro* differentiation followed by* in vivo* transplantation was performed in order to gain male gametes in advanced differentiation stages [24, 48] (Figure 1). Most often, pluripotent stem cells are induced in SSC-like stages* in vitro* and then transplanted into sterile mice testis [24] or ectopic, into the dorsal region of the mice, together with immature testicular cell suspension [44, 48]. It has been shown in several animal models that SSC-like cells are capable of recolonizing the testis [34, 35] and exhibit proper spermatogenesis [46]. A limiting step for stem cell replacement therapy of infertility could be the damaged somatic environment of the testis. If the somatic environment is damaged it is not receptive to SSC transplantation and thereby not able to restore patient fertility [57]. Ectopic cotransplantation of SSC with testicular cells might be a way to overcome this limitation but Yang et al. have reported that, although iPSC-derived germ cells could reconstitute seminiferous tubules and settle at basement membrane, no further differentiation was observed in reconstituted seminiferous tubules [44].

## 4. Stem Cell-Derived Oocytes: Current Knowledge and Future Perspectives

Stem cell-based strategies for ovarian regeneration and oocyte production have been proposed as future clinical therapies for treating infertility in women.

**Figure 2:**
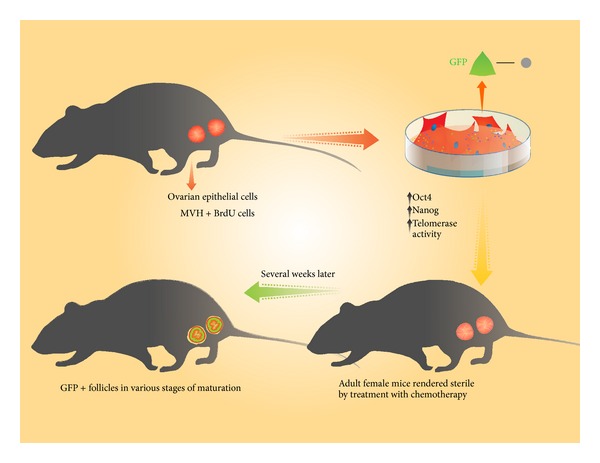
Ovarian stem cells:isolation and regenerative potential. Ovarian stem cells (MVH+BrdU+ cells) residing within the ovarian surface epithelium of neonatal and adult mice express high telomerase activity, Oct4, and Nanog and have a capacity to generate functional oocytes when transplanted back into sterile recipient mice.

There has been a long-persisting dilemma regarding the presence of ovarian stem cells in adult mammalian ovaries. Several research studies claimed that they have identified functional oogonial stem cells in the postnatal ovary of several different species including humans and now there is steadily increasing experimental evidence on their existence [58]. An important breakthrough was made by Zou and his coworkers who successfully established long-persisting pluripotent/multipotent ovarian stem cell lines in neonatal and adult mice [59]. They detected cells residing within the ovarian surface epithelium of neonatal and adult mice that were double positive for mouse vasa homologue (MVH) and DNA marker 5-bromodeoxyuridine (BrdU) confirming that these cells were of germ cell lineage and exhibited a replicative potential (Figure 2). With passage in culture, the cells isolated by Zou et al. were confirmed to have significant proliferative capacity and expressed high telomerase activity,* Oct4*, and* Nanog*. The cells were then marked using a retroviral vector bearing green fluorescent protein (GFP) before being directly delivered into the ovaries of adult female mice rendered sterile by treatment with chemotherapy. Importantly, GFP^+^ follicles in various stages of maturation were observed several weeks later in the ovaries of the conditioned mice indicating that isolated ovarian stem cells were capable of regenerating functional oocytes when transplanted back into sterile recipient mice [59] (Figure 2).

Recently, the work by White et al. has identified a rare population of mitotically active germ cells in human ovaries that can be purified and cultured* in vitro* to spontaneously form oocytes [60]. These cells, named as germ stem cells (GSCs), were isolated from reproductive-aged human ovaries using fluorescence-activated cell sorting (FACS) with an antibody against the carboxyl (−COOH) terminus of the germ cell-specific marker Ddx4, which is expressed on the cell surface of GSCs. Further, GSCs were capable of forming oocyte-like structures and incorporating into follicles under specific* in vitro* and* in vivo* conditions. This work highlights a unique potential to generate oocytes* in vitro* from isolated cells in reproductive-aged women who may have a depleted follicle pool from such genetic defects as fragile X-associated primary ovarian insufficiency. This recent advance, along with those described above, highlights the unique methodologies being developed to combat female-factor infertility representing a significant step towards the revolutionary idea of neo-oogenesis in reproductive-aged women through the isolation and characterization of germ stem cells.

However, despite the discovery of the potential germ stem cells in mammalian ovaries, it remains uncertain whether these cells exist and function in ovaries under physiological conditions. Liu et al. concluded that active meiosis, neo-oogenesis, and GSCs are unlikely to exist in normal, adult human ovaries [61]. Findings published by Zhang et al. contradict the results obtained by White et al. and Zhang et al. used fluorescent proteins to identify GSCs in the ovaries of mice, but these cells failed to divide or differentiate into oocytes [62]. The scientific community has questioned both the methods and significance of these studies. Supporters of postnatal de novo oogenesis disagree with the study conducted by Zhang et al. and state that the study investigated oocytes and not GSCs in their applied experimental setting; thus, the researchers never observed mitosis in Ddx4-positive cells since oocytes expressing cytoplasmic Ddx4 do not divide [63]. According to White et al., Ddx4 is found on the cell surface of GSCs and thus enables FACS-based isolation of living GSCs from adult mouse and human ovaries [60]. This is in contrast to the opinion of Zhang et al., who argue that Ddx4 is expressed only in the cytoplasm and not on the cell surface and hence FACS-based isolation of GSCs is problematic [64]. While the debate continues, only future experiments will help to clarify this issue.

In the meantime, several studies were published regarding the potential of pluripotent stem cells for differentiation into oocytes. Eguizabal and coworkers managed to generate haploid female cells from human pluripotent stem cells, but neither of them resembled an oocyte nor is predicated to possess a functional ooplasm capable of being fertilized [22]. However, the recent work by Hayashi et al. showed that mouse stem cells could be differentiated in an* in-vitro/in-vivo* system into oocyte-like cells that are capable of being fertilized by spermatozoa and generating normal progeny [65]. This outstanding advancement further shows the ability of pluripotent stem cells to differentiate into all cells of the adult organism [64]. Whether the work by Hayashi and colleagues can be adapted for human stem cells remains to be seen, but this advancement is a critical step forward in generating functional de novo oocytes from human iPSCs obtained from female patients rendered sterile by medical interventions, exposure to toxicants, or premature ovarian failure [64].

## 5. Conclusions

Pluripotent stem cells open new perspectives in the treatment of patients with azoospermia. Although the use of ESCs is connected with many ethical concerns, there are no ethical issues regarding the use of iPSCs. Moreover, ESCs are genetically unrelated to the patients, while it may be possible to get offspring with their own genetics by using iPSCs in derivation of functional male gametes.

The potential clinical applications of putative ovarian derived stem cells are apparent. The development of techniques to prolong the window of fertility for women has the ability to meet the needs of future populations and their delay in childbearing. If a viable source of oocyte production remains in infertile women with a reduced ovarian follicle pool, for example, due to chemotherapy or advanced age, the potential exists to restore fertility in these women. The identification of GSCs gives hope to these women and suggests the potential for fertility restoration. In future, the protocols for isolation and culture of GSCs must be optimized. In the meantime, production of germ cells from ESCs or iPSCs is another possible alternative for the treatment of infertility.

## Figures and Tables

**Table 1:** Characteristics of stem cells used in stem cell-based therapy of infertility.

ESCs	MSCs	Stem cell from extraembryonic tissues	iPSCs	Spermatogonial stem cells

Derived from inner cell mass of the blastocyst	Derived from bone marrow, adipose tissues, bone, Wharton's jelly, umbilical cord blood, and peripheral blood	Derived from amnion, chorion, placenta, and umbilical cord	Derived from somatic cells	Derived from testicular tissues

Pluripotent	Multipotent	Multipotent	Pluripotent	Pluripotent

These cells can differentiate into cell types of all three germ layers	These cells can differentiate into mesoderm-derived tissues (adipose tissues, bon, cartilage, and muscle)	These cells can differentiate into adipocytes, endothelial cells, hepatocytes, osteocytes, myocytes, and neurons	These cells can differentiate into cell types of all three germ layers	These cells can differentiate into cell types of all three germ layers

Prolonged proliferation	Degree of proliferation depends on the tissue from which these cells were isolated	Degree of proliferation depends on the tissue from which these cells were isolated	Prolonged proliferation	Difficult to be maintained in cultures

Indefinite self-renewal potential	Limited self-renewal	Limited self-renewal	Indefinite self-renewal potential	Self-renewal ability to go through numerous cell divisions while maintaining the undifferentiated state

High telomerase activity	Low telomerase activity	Low telomerase activity	High telomerase activity	High telomerase activity

Immortal; cell lines remain intact for long periods of time and produce endless numbers of cells	Production of limited number of cells	Production of limited number of cells	Immortal; cell lines remain intact for long periods of time and produce endless numbers of cells	—

These cells are not immune privileged	These cells have immunomodulatory characteristics	—	These cells are not immune privileged	These cells are not immune privileged

**Table 2:** Potential advantages and disadvantages of stem cells in regenerative medicine.

Stem cells	Advantages	Disadvantages
ESCs	Pluripotent; high telomerase activity	Ethical concerns; malignant potential; difficult to control; may require many steps to differentiate into desired cell type; immune rejection

MSCs	No ethical or moral concerns; low malignant potential; avoiding allogeneic immune rejection	Limited flexibility; multipotent; difficulty to be maintained in cell culture for long periods

Stem cell from extraembryonic tissues	No ethical or moral concerns; reducing risk of tumorigenicity	Limited flexibility; multipotent

iPSCs	No ethical or moral concerns; patient-specific cells	Use of viral vectors to introduce genes; malignant potential

Spermatogonial stem cells	No ethical or moral concerns	Relatively small numbers in testis; difficulty to be maintained in cultures; immune rejection

## References

[1] Boivin J, Bunting L, Collins JA, Nygren KG (2007). International estimates of infertility prevalence and treatment-seeking: potential need and demand for infertility medical care. *Human Reproduction*.

[2] Hull MGR, Glazener CMA, Kelly NJ (1985). Population study of causes, treatment, and outcome of infertility. *British Medical Journal*.

[3] Saunders DM, Mathews M, Lancaster PAL (1988). The Australian register: current research and future role. A preliminary report. *Annals of the New York Academy of Sciences*.

[4] Tan S-L, Doyle P, Campbell S (1992). Obstetric outcome of in vitro fertilization pregnancies compared with normally conceived pregnancies. *The American Journal of Obstetrics and Gynecology*.

[5] Goldenberg RL, Culhane JF, Iams JD, Romero R (2008). Epidemiology and causes of preterm birth. *The Lancet*.

[6] Schlegel PN (2009). Evaluation of male infertility. *Minerva Ginecologica*.

[7] Volarevic V, Ljujic B, Stojkovic P, Lukic A, Arsenijevic N, Stojkovic M (2011). Human stem cell research and regenerative medicine-present and future. *British Medical Bulletin*.

[8] Kolios G, Moodley Y (2013). Introduction to stem cells and regenerative medicine. *Respiration*.

[9] Rossant J (2001). Stem cells from the mammalian blastocyst. *Stem Cells*.

[10] de Miguel MP, Fuentes-Julián S, Alcaina Y (2010). Pluripotent stem cells: origin, maintenance and induction. *Stem Cell Reviews and Reports*.

[11] Evans MJ, Kaufman MH (1981). Establishment in culture of pluripotential cells from mouse embryos. *Nature*.

[12] Ratajczak MZ, Zuba-Surma E, Kucia M, Poniewierska A, Suszynska M, Ratajczak J (2012). Pluripotent and multipotent stem cells in adult tissues. *Advances in Medical Sciences*.

[13] Augello A, Kurth TB, de Bari C (2010). Mesenchymal stem cells: a perspective from in vitro cultures to in vivo migration and niches. *European Cells and Materials*.

[14] Bruder SP, Jaiswal N, Haynesworth SE (1997). Growth kinetics, self-renewal, and the osteogenic potential of purified human mesenchymal stem cells during extensive subcultivation and following cryopreservation. *Journal of Cellular Biochemistry*.

[15] Prockop DJ (1997). Marrow stromal cells as stem cells for nonhematopoietic tissues. *Science*.

[16] Friedenstein AJ, Chailakhjan RK, Lalykina KS (1970). The development of fibroblast colonies in monolayer cultures of guinea-pig bone marrow and spleen cells. *Cell and Tissue Kinetics*.

[17] Barzilay R, Melamed E, Offen D (2009). Introducing transcription factors to multipotent mesenchymal stem cells: making transdifferentiation possible. *Stem Cells*.

[18] Marcus AJ, Woodbury D (2008). Fetal stem cells from extra-embryonic tissues: do not discard: Stem Cells Review Series. *Journal of Cellular and Molecular Medicine*.

[19] Takahashi K, Yamanaka S (2006). Induction of pluripotent stem cells from mouse embryonic and adult fibroblast cultures by defined factors. *Cell*.

[20] Malik N, Rao MS (2013). A review of the methods for human iPSC derivation. *Methods in Molecular Biology*.

[21] Xu XL, Yi F, Pan HZ (2013). Progress and prospects in stem cell therapy. *Acta Pharmacologica Sinica*.

[22] Eguizabal C, Montserrat N, Vassena R (2011). Complete meiosis from human induced pluripotent stem cells. *Stem Cells*.

[23] Easley CA, Phillips BT, McGuire MM (2012). Direct differentiation of human pluripotent stem cells into haploid spermatogenic cells. *Cell Reproduction*.

[24] Zhu Y, Hu HL, Li P (2012). Generation of male germ cells from induced pluripotent stem cells (iPS cells):an in vitro and in vivo study. *Asian Journal of Andrology*.

[25] Kita K, Watanabe T, Ohsaka K (2007). Production of functional spermatids from mouse germline stem cells in ectopically reconstituted seminiferous tubules. *Biology of Reproduction*.

[26] De Kretser DM, Baker HWG (1999). Infertility in men: recent advances and continuing controversies. *The Journal of Clinical Endocrinology and Metabolism*.

[27] Kanatsu-Shinohara M, Lee J, Inoue K (2008). Pluripotency of a single spermatogonial stem cell in mice. *Biology of Reproduction*.

[28] McLaren A (2003). Primordial germ cells in the mouse. *Developmental Biology*.

[29] McLean DJ (2005). Spermatogonial stem cell transplantation and testicular function. *Cell and Tissue Research*.

[30] Nolte J, Michelmann HW, Wolf M (2010). PSCDGs of mouse multipotent adult germline stem cells can enter and progress through meiosis to form haploid male germ cells in vitro. *Differentiation*.

[31] Guan K, Nayernia K, Maier LS (2006). Pluripotency of spermatogonial stem cells from adult mouse testis. *Nature*.

[32] Hamra FK, Schultz N, Chapman KM (2004). Defining the spermatogonial stem cell. *Developmental Biology*.

[33] Izadyar F, Wong J, Maki C (2011). Identification and characterization of repopulating spermatogonial stem cells from the adult human testis. *Human Reproduction*.

[34] Brinster RL (2007). Male germline stem cells: from mice to men. *Science*.

[35] Hermann BP, Sukhwani M, Winkler F (2012). Spermatogonial stem cell transplantation into rhesus testes regenerates spermatogenesis producing functional sperm. *Cell Stem Cell*.

[36] Hübner K, Fuhrmann G, Christenson LK (2003). Derivation of oocytes from mouse embryonic stem cells. *Science*.

[37] Nayernia K, Nolte J, Michelmann HW (2006). In vitro-differentiated embryonic stem cells give rise to male gametes that can generate offspring mice. *Developmental Cell*.

[38] Zhao X-Y, Li W, Lv Z (2010). Viable fertile mice generated from fully pluripotent iPS cells derived from adult somatic cells. *Stem Cell Reviews and Reports*.

[39] Clark AT, Bodnar MS, Fox M (2004). Spontaneous differentiation of germ cells from human embryonic stem cells in vitro. *Human Molecular Genetics*.

[40] Kee K, Gonsalves JM, Clark AT, Reijo Pera RA (2006). Bone morphogenetic proteins induce germ cell differentiation from human embryonic stem cells. *Stem Cells and Development*.

[41] Mikkola M, Olsson C, Palgi J (2006). Distinct differentiation characteristics of individual human embryonic stem cell lines. *BMC Developmental Biology*.

[42] Chen H-F, Kuo H-C, Chien C-L (2007). Derivation, characterization and differentiation of human embryonic stem cells: comparing serum-containing versus serum-free media and evidence of germ cell differentiation. *Human Reproduction*.

[43] Tilgner K, Atkinson SP, Golebiewska A, Stojković M, Lako M, Armstrong L (2008). Isolation of primordial germ cells from differentiating human embryonic stem cells. *Stem Cells*.

[44] Yang S, Bo J, Hu H (2012). Derivation of male germ cells from induced pluripotent stem cells in vitro and in reconstituted seminiferous tubules. *Cell Proliferation*.

[45] Li P, Hu H, Yang S (2013). Differentiation of induced pluripotent stem cells into male germ cells *in vitro* through embryoid body formation and retinoic acid or testosterone induction. *BioMed Research International*.

[46] Hayashi K, Ohta H, Kurimoto K, Aramaki S, Saitou M (2011). Reconstitution of the mouse germ cell specification pathway in culture by pluripotent stem cells. *Cell*.

[47] Ohinata Y, Ohta H, Shigeta M, Yamanaka K, Wakayama T, Saitou M (2009). A signaling principle for the specification of the germ cell lineage in mice. *Cell*.

[48] Cai H, Xia X, Wang L (2013). In vitro and in vivo differentiation of induced pluripotent stem cells into male germ cells. *Biochemical and Biophysical Research Communications*.

[49] Zhu S, Li W, Zhou H (2010). Reprogramming of human primary somatic cells by OCT4 and chemical compounds. *Cell Stem Cell*.

[50] Bowles J, Knight D, Smith C (2006). Retinoid signaling determines germ cell fate in mice. *Science*.

[51] Koubova J, Menke DB, Zhou Q, Cape B, Griswold MD, Page DC (2006). Retinoic acid regulates sex-specific timing of meiotic initiation in mice. *Proceedings of the National Academy of Sciences of the United States of America*.

[52] Griswold MD (1998). The central role of Sertoli cells in spermatogenesis. *Seminars in Cell and Developmental Biology*.

[53] Byskov AG, Fenger M, Westergaard L, Andersen CY (1993). Forskolin and the Meiosis Inducing Substance synergistically initiate meiosis in fetal male germ cells. *Molecular Reproduction and Development*.

[54] Eguizabal C, Boyano MD, Díez-Torre A (2007). Interleukin-2 induces the proliferation of mouse primordial germ cells in vitro. *International Journal of Developmental Biology*.

[55] Kanatsu-Shinohara M, Inoue K, Ogonuki N (2007). Leukemia inhibitory factor enhances formation of germ cell colonies in neonatal mouse testis culture. *Biology of Reproduction*.

[56] de Rooij DG (2006). Regulation of spermatogonial stem cell behavior in vivo and in vitro. *Animal Reproduction*.

[57] Easley CA, Simerly CR, Schatten G (2013). Stem cell therapeutic possibilities: future therapeutic options for male-factor and female-factor infertility?. *Reproductive Biomedicine Online*.

[58] Virant-Klun I, Stimpfel M, Skutella T (2011). Ovarian pluripotent/multipotent stem cells and in vitro oogenesis in mammals. *Histology and Histopathology*.

[59] Zou K, Yuan Z, Yang Z (2009). Production of offspring from a germline stem cell line derived from neonatal ovaries. *Nature Cell Biology*.

[60] White YAR, Woods DC, Takai Y, Ishihara O, Seki H, Tilly JL (2012). Oocyte formation by mitotically active germ cells purified from ovaries of reproductive-age women. *Nature Medicine*.

[61] Liu Y, Wu C, Lyu Q (2007). Germline stem cells and neo-oogenesis in the adult human ovary. *Developmental Biology*.

[62] Zhang H, Zheng W, Shen Y (2012). Experimental evidence showing that no mitotically active female germline progenitors exist in postnatal mouse ovaries. *Proceedings of the National Academy of Sciences of the United States of America*.

[63] Yong E (2012). Ovarian stem cell debate. *The Scientist*.

[64] Evron A, Blumenfeld Z (2013). Ovarian stem cells-the pros and cons. *Clinical Medicine Insights Reproductive Health*.

[65] Hayashi K, Ogushi S, Kurimoto K (2012). Offspring from oocytes derived from in vitro primordial germ cell-like cells in mice. *Science*.

